# Improved Controlled Release and Brain Penetration of the Small Molecule S14 Using PLGA Nanoparticles †

**DOI:** 10.3390/ijms22063206

**Published:** 2021-03-22

**Authors:** Vanesa Nozal, Elisa Rojas-Prats, Inés Maestro, Carmen Gil, Daniel I. Perez, Ana Martinez

**Affiliations:** 1 Centro de Investigaciones Biológicas Margarita Salas-CSIC, Ramiro de Maeztu 9, 28040 Madrid, Spain; vanesanozal@cib.csic.es (V.N.); ely.rp.91@gmail.com (E.R.-P.); ines.maestro@cib.csic.es (I.M.); carmen.gil@csic.es (C.G.); 2 Centro de Investigación Biomédica en Red de Enfermedades Neurodegenerativas (CIBERNED), Instituto de Salud Carlos III, 28031 Madrid, Spain

**Keywords:** nanoparticle, controlled release, brain penetration, phosphodiesterase 7 inhibitor, PLGA, nanoprecipitation

## Abstract

Phosphodiesterase 7 (PDE7) is an enzyme responsible for the degradation of cyclic adenosine monophosphate (cAMP), an important cellular messenger. PDE7’s role in neurotransmission, expression profile in the brain and the druggability of other phosphodiesterases have motivated the search for potent inhibitors to treat neurodegenerative and inflammatory diseases. Different heterocyclic compounds have been described over the years; among them, phenyl-2-thioxo-(*1H*)-quinazolin-4-one, called S14, has shown very promising results in different in vitro and in vivo studies. Recently, polymeric nanoparticles have been used as new formulations to target specific organs and produce controlled release of certain drugs. In this work, we describe poly(lactic-co-glycolic acid) (PLGA)-based polymeric nanoparticles loaded with S14. Their preparation, optimization, characterization and in vivo drug release profile are here presented as an effort to improve pharmacokinetic properties of this interesting PDE7 inhibitor.

## 1. Introduction

Phosphodiesterases (PDEs) are key enzymes involved in the hydrolysis of adenine and guanosine 3′,5′-cyclic monophosphates (cAMP and cGMP), which comprise over 11 subfamilies with different expression patterns throughout the human body and different selectivity for cAMP and cGMP [1]. PDE4, PDE7 and PDE8 are cAMP-specific and degrade it to its inactive 5′-monophosphate form. This process has a crucial importance due to the central role of cAMP in a variety of cellular responses, naming gene transcription, mitochondrial homeostasis, neurotransmission, cell migration, proliferation or cell death [2]. Besides, increased cAMP levels control anti-inflammatory and immunosuppressive processes. Among the cAMP-specific PDEs, there have been tremendous efforts to understand the role of PDE7 in human diseases and to overcome adverse effects associated with the widely studied PDE4 inhibitors, mainly the emetic effect [3]. PDE7 has two closely related isoforms, PDE7A and PDE7B, and it is widely expressed in the brain having a role in neurotransmission. Moreover, the expression of PDE7 isoforms is also described in peripheral blood cells. Thus, inhibitors of these isoforms have been described as potential treatments for neurodegenerative and inflammatory diseases such as multiple sclerosis or Alzheimer’s and Parkinson’s disease (PD) [4,5].

**Figure 1:**
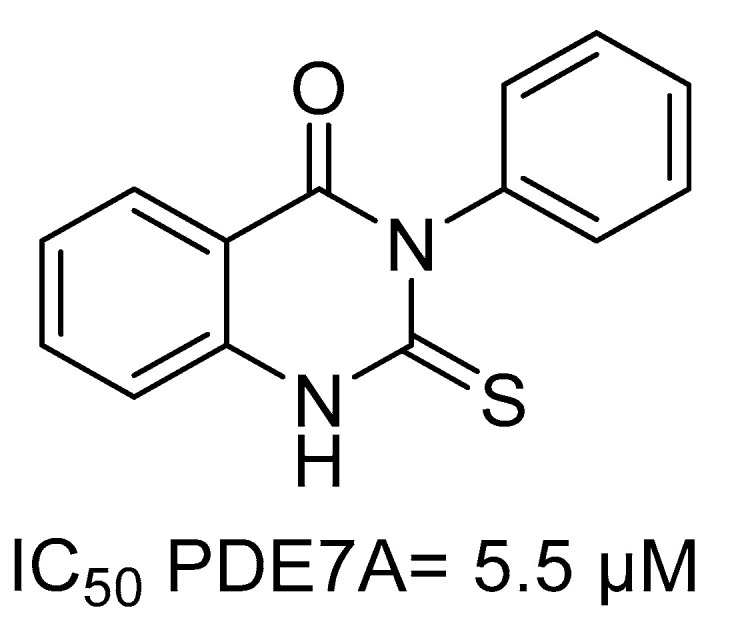
Chemical structure of S14, a selective PDE7 inhibitor.

Although the specific function of PDE7 in PD is not fully understood, recent studies show the key role of genetic inhibition for neuroprotection and neuroinflammation reduction in two different models of dopaminergic neurons loss [6,7]. Since reduced cAMP levels are related to microglia inflammation, treatment with PDE7 inhibitors could bring a significant additional benefit for the patients. Among the different PDE7 inhibitors reported in literature [8], phenyl-2-thioxo-(*1H*)-quinazolin-4-one, named S14 (Figure 1) [9], has shown a great potential to treat PD. Quinazoline S14 demonstrated efficacy to rescue dopaminergic cell death and to reduce glial activation in a PD model. Importantly, this effect was proved to be mediated by cAMP elevation [10]. In addition to reduce neuroinflammation, PDE7 inhibition by S14 promoted neurogenesis in the hippocampus and subventricular zone in vitro and in vivo [11] and dopaminergic neurogenesis [12]. Moreover, as validation of PDE7 was also achieved by treatment of specific siRNA in hemiparkinsonian mice [7], the value of PDE7 as pharmacological target for PD was confirmed and the potential of S14 as disease-modifying treatment reinforced.

For these reasons, and in order to boost the advance of S14 to human clinical trials, we here propose the development of a novel formulation to improve the pharmacokinetic profile of the compound. In this sense, nanoparticles (NPs) have emerged as a useful tool to address some of the problems associated with conventional therapeutic agents such as unspecific distribution, rapid metabolism or low bioavailability [13]. Over the past years, organic nanoparticles, and especially polymeric nanoparticles and liposomes, have been used to load drugs and improve their solubility, ameliorate their biodistribution and prolong their circulating half-life. All these efforts resulted in the approval of diverse nanoparticles-containing formulations by the regulatory agencies [14]. In our case, we have chosen poly(lactic-co-glycolic acid) (PLGA) nanoparticles to load S14 and improve its pharmacokinetic profile based on their physicochemical properties and the good results described in the literature for the release of certain drugs [15].

Different preparation methods and conditions were tested in order to find the best procedure, variating surfactants, solvents, drug load and production methodology. Once the best one was selected, absence of toxicity and improvement in the pharmacokinetic profile of S14-loaded PLGA nanoparticles was finally demonstrated testing the new formulation in vitro and in vivo. Finally, preliminary scale-up experiments were performed to confirm the feasibility of the large-scale process for future clinical applications.

## 2. Results and Discussion

### 2.1. Nanoparticles Preparation and Optimization Process

Some successful examples of biodegradable polymeric nanoparticle formulations have recently been approved by the Food and Drug Administration (FDA) such as polyethylene glycol (PEG), polylactic acid (PLA) or polyvinylpyrrolidone/ethyl cellulose [16]. Among them, PLGA copolymer is the most used due to the absence of toxicity owing to its degradation into simple glycolic and lactic acids which are eliminated by the normal metabolic pathways [17]. These evidences motivated us to choose PLGA as polymer for S14-loaded nanoparticles preparation. Different methodologies have been well described to prepare polymeric nanoparticles depending on their clinical applications and the properties of the drug to be entrapped, like emulsification-solvent evaporation, emulsification-solvent diffusion, dialysis or nanoprecipitation [18]. In order to encapsulate hydrophobic drugs, a variant of the emulsification-solvent evaporation methodology called oil-in-water or single emulsion and nanoprecipitation are the most widely used methods. In the present work, both techniques were evaluated for the encapsulation of S14.

**Figure 2:**
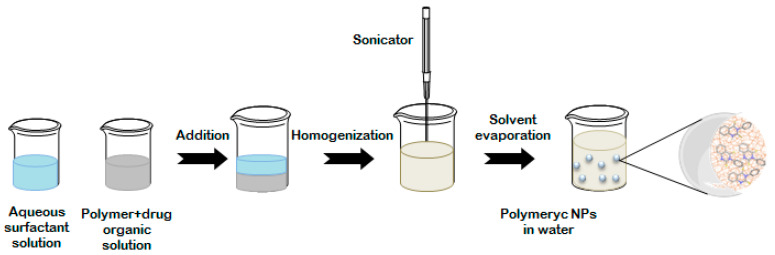
Schematic representation of the single-emulsion methodology for PLGA nanoparticles preparation. PLGA: poly(lactic-co-glycolic acid); NPs: nanoparticles.

As a first approach for the preparation of the S14-loaded nanoparticles, single-emulsion method was implemented (Figure 2) [19]. In this case, the polymer and the drug are dissolved into the same water-immiscible organic solvent such as chloroform or ethyl acetate, and different surfactants are used to prevent nanoparticles aggregation.

In order to optimize the method, six different formulations were prepared (NP-1.1 to NP-1.6), summarized in Table 1. The type and concentration of the polymer in the organic phase were fixed (PLGA 50:50), while the nature of the surfactant and its concentration in the aqueous phase were varied. Cellulose nanocrystals (CNC), Poloxamer 188 (P188 or Pluronic F68) and polyvinyl alcohol (PVA) were used due to their good properties as stabilizers based on different studies reported previously in the literature [20]. In addition, cellulose, P188 and PVA are approved by the FDA for clinical applications [21,22], PVA being one of the most used surfactants for PLGA nanoparticles preparation [23].

The effect of the initial amount of the drug on the preparation of PLGA nanoparticles was also evaluated using 10 mg and 20 mg.

As shown in Table 1, those formulations prepared in the presence of different concentrations of poloxamer as surfactant (NP-1.2, NP-1.3 and NP-1.4) failed to provide nanoparticles as the polymer precipitated and no emulsion was formed, while a low number of nanoparticles was obtained in the case of CNC and PVA using 10 mg of S14 (NP-1.1 and NP-1.5, respectively). Regarding the initial amount of the drug used, the final quantity of nanoparticles was slightly increased when 20 mg of S14 were used (NP-1.6) as a consequence of a greater drug encapsulation. However, this methodology failed when using 30 mg of S14 due to the precipitation of both the polymer and the drug in the same experimental conditions.

**Figure 3:**
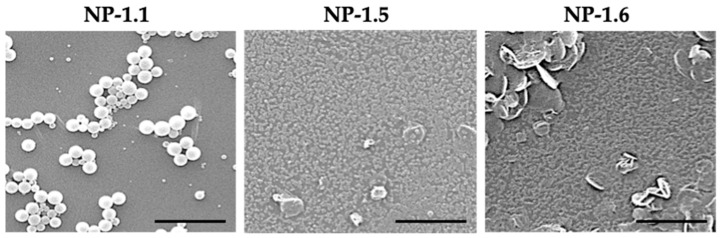
SEM images of S14-loaded PLGA nanoparticles prepared by single-emulsion methodology. Scale bars: 5 μm.

In order to study the morphology of the nanoparticles, samples were analyzed by Scanning Electron Microscopy (SEM) (Figure 3) [24]. This technique is widely used to characterize nanoparticles in size, shape and surface by a direct visualization of the sample after coating with a thin layer of conductive material. 

Considering shape and surface, S14-loaded nanoparticles have a quasi-spherical shape and smooth surface for all formulations. After analysis of SEM images, we concluded that the use of CNC gave large nanoparticles in size, with an approximate average of 1 μm (NP-1.1), while more regular and smaller NPs were obtained in presence of PVA (NP-1.5 and NP-1.6). However, the final number of nanoparticles was too low in all cases, ranging from 10 to 27 mg, limiting the use of this methodology to encapsulate S14. 

**Figure 4:**
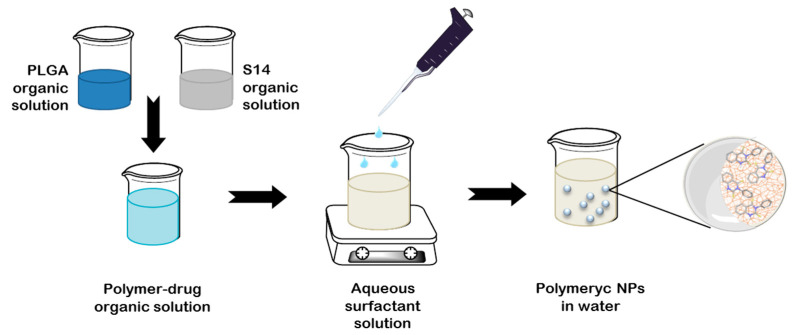
Schematic representation of the nanoprecipitation methodology.

As an effort to improve S14-loaded nanoparticles regarding size, shape and reproducibility, nanoprecipitation methodology was assayed (Figure 4). This method, firstly described by Fessi et al. [25], is a one-step procedure based on the interfacial deposition of the PLGA due to the rapid diffusion of the organic solvent to the aqueous phase. The drug and the polymer are dissolved in the same water-miscible organic solvent, tetrahydrofuran (THF) in this case, and PVA was selected as surfactant because it gave the best outcome in the previous methodology.

Nanoprecipitation methodology was performed by duplicate using increasing amounts of the drug (10, 20, 30 and 40 mg), while surfactant and polymer concentrations were invariable according to previously optimized conditions (Table 2) [26]. In all cases, this method was able to provide a good number of nanoparticles (from 32 to 78 mg), which increased with the amount of the drug used with great reproducibility. Furthermore, lower concentration of the polymer solution was required compared to single-emulsion methodology. Thus, only 50 mg of PLGA were needed for nanoparticles preparation, while 250 mg of the same polymer were used when single-emulsion methodology was implemented. Finally, we concluded that the top limit of nanoparticles drug loading with these conditions reached 40 mg, as the same proportion of components failed to give a proper number of nanoparticles when using 50 mg of the drug, once again due to the precipitation of the polymer and the drug.

**Figure 5:**
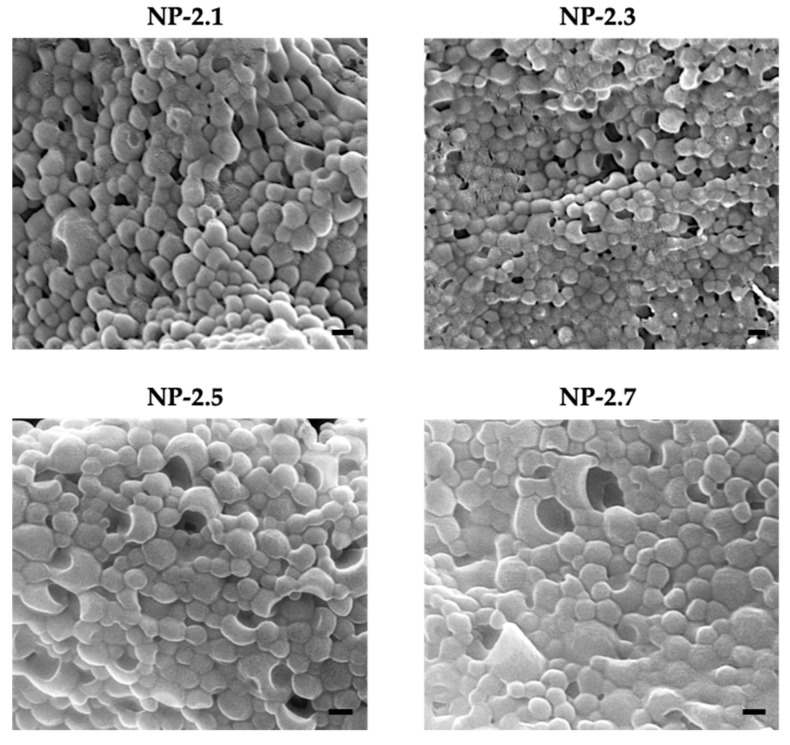
Representative SEM images of S14-loaded PLGA nanoparticles prepared by nanoprecipitation methodology. Scale bars: 100 nm.

All the samples were also analyzed by SEM to determine the morphology of the nanoparticles. The different images obtained are represented in Figure 5. 

As it can be seen, nanoparticles have a mostly spherical and smooth shape with an approximate size of 100 nm. Besides, all formulations present similar aspect without being influenced by their S14-load.

Since nanoprecipitation resulted in the most efficient methodology for the preparation of S14-loaded PLGA-NPs in terms of reproducibility, ease of procedure, use of materials and initial drug-loading, we chose this methodology for the encapsulation of S14 and the improvement of its pharmacokinetic profile. Thus, different properties of these nanoparticles such as drug loading, size and polydispersity, in addition to in vitro and in vivo toxicity, were evaluated in further studies in order to complete the analysis of this interesting formulation.

### 2.2. Encapsulation Efficiency (EE) and Drug-Loading Capacity (LC)

The efficacy of prepared PLGA nanoparticles to entrap S14 is an important parameter that must be calculated when preparing nanoparticle systems for drug delivery. This can be measured by two different parameters: encapsulation efficiency (EE) and drug-loading capacity (LC) [27]. In this study, both values were quantified by a direct method using High-Performance Liquid Chromatography (HPLC) analysis [28] and expressed according to Equations (1) and (2) described in the Materials and Methods section. In the first place, a calibration curve of free S14 was used to correlate the peak area with the drug concentration (Figure S1). Then, the entrapped S14 was directly measured after breakage of the nanoparticles in acetonitrile, which was able to break the nanoparticles while dissolving the drug. The different obtained formulations are summarized in Table 3.

The EE for our S14-loaded PLGA-NPs is in the range between 65% and 88%, while the LC is around 20% to 47%. The highest values were obtained for formulations NP-2.7 and NP-2.8, where 40 mg of S14 were used for nanoparticles preparation. These results are similar to other drug-loaded PLGA nanoparticles reported in the literature where the mean encapsulation efficiency is between 60% and 70% and improves the described drug-loading capacity which is usually low (around 1%) [29]. These results reinforced the choice of nanoprecipitation for nanoparticles preparation, as low quantity of the formulation needs to be administered in order to obtain the desired doses due to the high EE and LC of the nanoparticles.

### 2.3. Size, Polydispersity Index and Zeta Potential

In order to determine the size of the S14-loaded nanoparticles more accurately as well as to assess degree of dispersity of the samples, Dynamic Light Scattering (DLS) technique was used [30]. This technique is based on the Brownian motion of particles in solution, which directly correlates with particle size through the Stokes–Einstein equation. To this purpose, formulations NP-2.1, NP-2.3, NP-2.5 and NP-2.7 were selected as representative of each trial.

**Figure 6:**
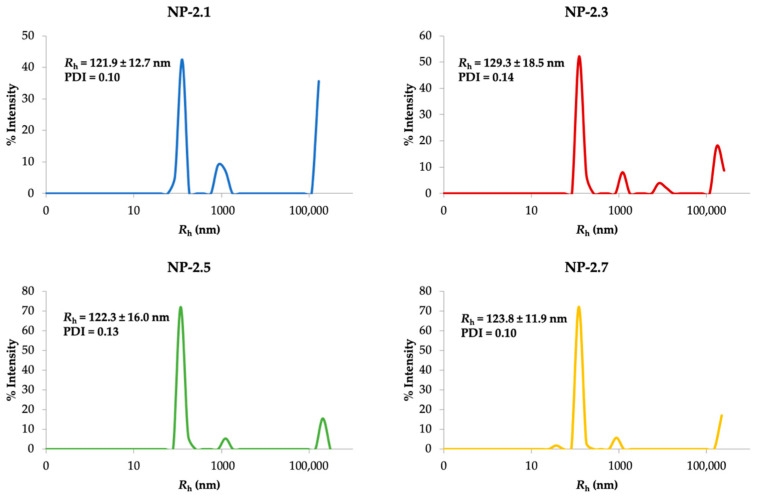
Representative particle size distribution of S14-loaded PLGA-NPs. Results are shown as the mean of 40 measures ± standard deviation (SD). PDI refers to polydispersity of NPs.

In our study, the hydrodynamic radius (*R*_h_) obtained for all formulations is in the range between 121 and 129 nm (Figure 6), which is similar to other nanoparticles in pre-clinical studies for drug delivery into the brain [31]. Additionally, the polydispersity index (PDI) was calculated as it refers to the homogeneity of the particle solution. In all cases, the PDI values obtained (<0.15) indicate a monodisperse particle suspension with a narrow size distribution. Besides, the initial amount of S14 in the preparation process does not seem to influence the size and dispersity of the nanoparticles.

Furthermore, zeta potential was measured for the different formulations to determine the surface properties of the nanoparticles, and hence, stability of nanoparticles in suspension [32]. This value was determined by laser Doppler anemometry. The zeta potential values obtained are around −11.6 ± 0.2 mV, and this negative surface charge is attributed to the ionization of carboxyl end groups of the polymer.

Regarding these results, nanoprecipitation methodology provided not only good size and shape nanoparticles with high reproducibility but also high drug-loading nanoparticles in a simple and fast way.

Finally, as the different nanoparticles are very similar in size, PDI and zeta potential, formulations NP-2.5 to NP-2.8 are the best ones as greater amounts of the drug are encapsulated in the same preparation conditions. These formulations were chosen to test the in vitro and in vivo profile of the nanoparticles. 

### 2.4. In Vitro Viability Studies of Nanoparticles Formulations

**Figure 7:**
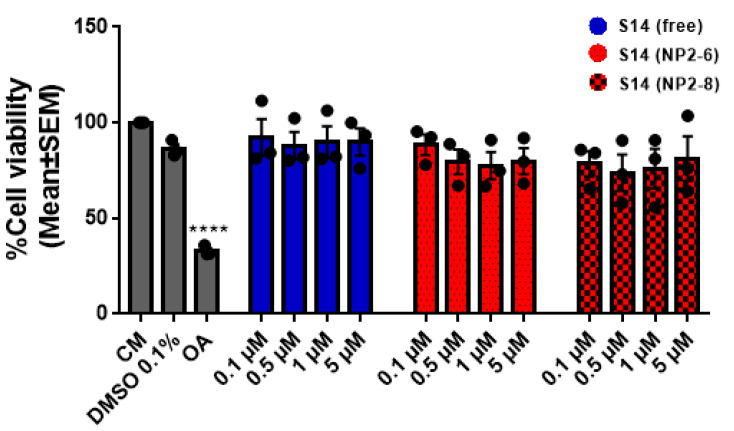
Cell viability of SH-SY5Y treated with increasing doses of free S14 and S14-loaded NPs for 24 h. Complete Media (CM) was used as vehicle for NPs, DMSO was used as vehicle for free S14 and Okadaic acid (OA) was used as internal control. Data represent the mean ± SEM of 3 different experiments. (**** *p* < 0.0001 significantly different from CM).

The next step in the feasibility study of these new formulations for S14 was to assess their effect on cell viability. Thus, human neuroblastoma cell line SH-SY5Y was selected as the main indications of S14 are central nervous system diseases. Cells were treated with increasing concentrations of free S14 and encapsulated S14 in two different formulations (NP-2.6 and NP-2.8) using equivalent doses of the drug from 0.1 µM to 5 µM (Figure 7). The colorimetric assay using 3-(4,5-dimethylthiazol)-2-yl)-2,5-diphenylte- trazolium bromide (MTT assay) was selected to assess the cell metabolic activity that reflects the number of viable cells. Okadaic acid (OA), a potent phosphatases inhibitor with toxic effects for the cells, was employed as a test for the MTT assay, showing a clear decrease on cell viability. In the same conditions, nanoparticles loaded with S14 have no effect on cell survival, showing a safe profile for further in vivo studies.

### 2.5. In Vivo Controlled Release

One of the most interesting advantages of drug-loaded nanoparticles is the controlled release of the drug over time and improvement of brain-to-plasma membrane permeability. In vivo experiments were set up in order to compare the concentration of the drug in free form vs. the drug-loaded nanoparticles. To conduct the experiments, nanoparticles formulation NP-2.5 was selected based on the entrapped efficiency and experimental availability. In our case, albino mice were used to test the pharmacokinetic profile of both formulations over 24 h after administration. Thereby, animals were administered with free S14 and S14-loaded nanoparticles (NP-2.5) in phosphate buffered saline at a dose of 10 mg/kg by oral route.

**Figure 8:**
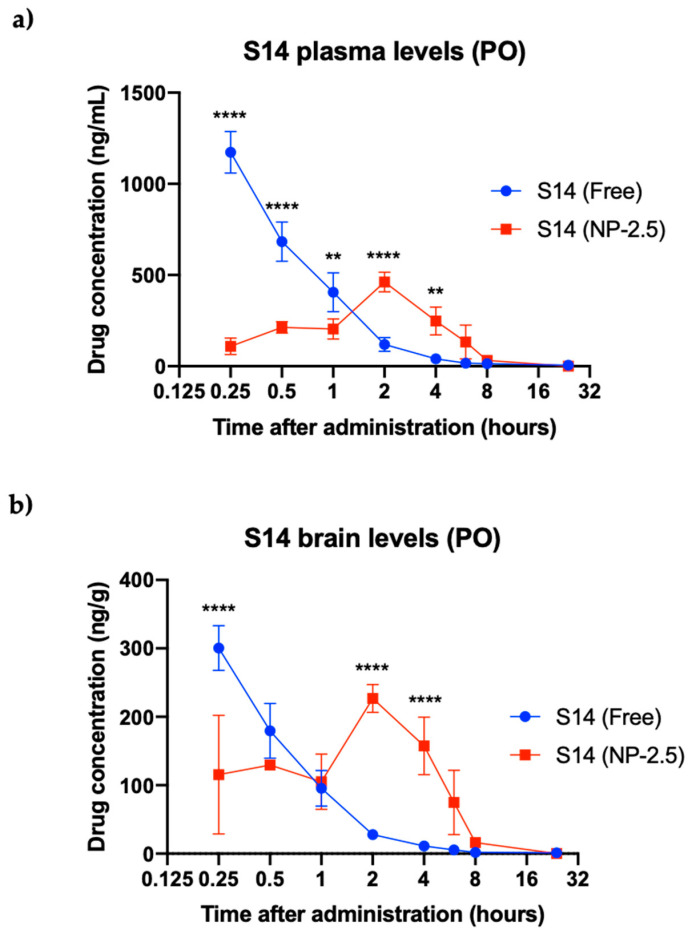
In vivo release experiments for free S14 vs. S14-loaded PLGA nanoparticles. S14 plasma levels (**a**) and brain levels (**b**) after oral administration (10 mg/kg). (** *p* < 0.005, **** *p* < 0.0001 significantly different from free S14).

All the mice presented normal behavior up to 24 h after administration with each formulation without any toxic effect. After monitoring the animals and analyzing plasma and brain concentration levels, we observed an improvement in the pharmacokinetic profile of S14 when loaded and administered in the nanoparticle form compared to the free drug administration (Figure 8). Maximum concentration levels were reached 15 min after administration for free S14 and 2 h for nanoparticles. In addition, in those animals where nanoparticles were administered, the drug levels in the brain lasted longer over the course of the experiment. On the contrary, free-S14-treated animals showed higher concentrations at a very short time (only 15 min) and a fast decrease in drug concentration. 

Penetrance of S14 to the brain was also improved when the drug-loaded nanoparticles were administered: plasma-to-brain ratios were 1.7:1, whereas in the free-drug-treated animals this ratio was 4.4:1 (Table S1), indicating that the nanoparticle formulation NP-2.5 improves not only the controlled release of the drug into the brain but also reduces its clearance in plasma. Similar results are reported in the literature where PLGA nanoparticles enhance delivery of dopamine into the brain of parkinsonian rats and protect dopamine from rapid peripheral metabolism [33].

### 2.6. Scale-Up Procedure of Nanoparticles

Finally, we focused in the preliminary scale-up process of S14 encapsulation since primary studies have shown that the drug presents no cardiovascular, respiratory and central nervous system toxicity as well as negative results in the genotoxicity studies [34]. For this reason, we increased five times the mass of the drug during nanoparticles preparation as well as the mass of PLGA and PVA. This time, the same S14/PLGA/PVA (1:1.25:10) mass ratio as NP-2.7 and NP-2.8 were used as the top limit of nanoparticles preparation, with the selected conditions reaching 40 mg. As result, we were able to increase the mass of the drug up to 200 mg for each preparation without affecting the final outcome of the formulations (Table 4). Above this value, the drug and the polymer precipitate during nanoparticles preparation.

It is not rare that some advantageous characteristics are lost during an escalation process of a laboratory method. However, in this case, shape of the S14-loaded PLGA-NPs were maintained, showing once again a smooth surface and almost-spherical shape by SEM analysis (Figure S2a). Furthermore, in all cases nanoparticles exhibit a hydrodynamic radius around 95 nm with PDI values that agree with a monodisperse particle suspension by DLS determinations (Figure S2b).

In the same way, the efficacy of these nanoparticles to encapsulate S14 was also calculated and expressed by both the encapsulation efficiency and drug-loading capacity parameters. This time, the EE% ranges from 59% to 73% with a mean LC% of 57% (Table S2).

Finally, we can conclude that the scale-up process for S14-loaded PLGA-NPs has successfully provided a larger number of nanoparticles with good properties and EE% values, and, in addition with its improved pharmacokinetic profile, the potential of S14-loaded nanoparticles for its future industrial escalation and application in clinical trials is confirmed.

## 3. Materials and Methods

### 3.1. Materials

Poly(lactic-co-glycolic acid) copolymer (PLGA, LACTEL^®^ Absorbable Polymers, Birmingham, USA), inherent viscosity range: 0.95–1.20 dL/g in HFIP, ester terminated (nominal), Part.#B6010-4P) with a 50:50 ratio (PLA/PGA) was used as biodegradable polymer for the nanoparticle preparation. Cellulose nanocrystals (CNC, samples kindly provided by Dr. Nicoletta Resignano, Instituto de Ciencia y Tecnología de Polímeros-CSIC, Madrid, Spain), poloxamer 188 (Kolliphor^®^ P188, BASF, Ludwigshafen, Germany) and polyvinyl alcohol (PVA, Sigma Aldrich, Madrid, Spain), Mw 31,000–50,000, 87–89% hydrolyzed, Cat. Number 363073) were used as surfactants. Ethyl acetate (EtOAc), tetrahydrofuran (THF), acetonitrile and deionized water were purchased from different commercial sources and used as solvents.

### 3.2. S-14-Loaded PLGA Nanoparticles Preparation

S14-loaded PLGA nanoparticles were prepared using two different methodologies: emulsification-solvent evaporation (single emulsion) and nanoprecipitation [18].

#### 3.2.1. Single Emulsion

For single emulsion, 125 mg of PLGA copolymer were dissolved in 5 mL of EtOAc, applying 2 h of magnetic stirring at room temperature. This solution was mixed with an organic solution containing 10 or 20 mg of the drug in 5 mL of EtOAc. The mixture was then emulsified with 20 mL of an aqueous solution containing the surfactant (0.5% *w*/*v* CNC, 1–5% *w*/*v* Poloxamer 188 or 2% *w*/*v* PVA) using a tip sonicator (Vibra-Cell^TM^ VC750, Bioblock Scientific, Madrid, Spain) for 30 min. The resulting emulsion was transferred in 200 mL of surfactant aqueous solution (0.05% *w*/*v* CNC, 0.1–0.5% *w*/*v* poloxamer 188 or 0.2% *w*/*v* PVA) and was magnetically stirred for 30 min at room temperature and concentrated to 50% of the initial volume under reduced pressure. Finally, NPs were collected by centrifugation (Sorvall RC-5C Centrifuge, SS-34 Rotor) at 15,000 rpm for 10 min and washed out three-to-four times with deionized water, frozen at −80 °C and lyophilized using a fast-freeze flask (Labconco Corp. 750 mL, Cat.#10033692, Kansas, USA) at −45 °C and 3.2 × 10^−2^ mbar pressure.

#### 3.2.2. Nanoprecipitation

In this case, 50 mg of PLGA copolymer were dissolved in 5 mL of THF with magnetic stirring for 2 h at room temperature and mixed with 10–40 mg of the drug in 5 mL of THF. The mixture was added dropwise (600 μL/min) over 20 mL of aqueous solution containing 2% *w*/*v* PVA. The resulting emulsion was stirred for 2–3 h at room temperature to eliminate the solvent. Finally, NPs were treated and collected as previously described. For the escalation process, 250 mg of PLGA and 200 mg of S14 were dissolved in 50 mL of THF and the mixture was added dropwise over 100 mL of the 2% *w*/*v* surfactant aqueous solution.

### 3.3. Characterization of Nanoparticles

#### 3.3.1. Scanning Electron Microscopy (SEM)

The morphology of the S14-loaded nanoparticles was analyzed by Scanning Electron Microscopy (SEM) using a JEOL JSM 7699F microscope (JEOL, Tokyo, Japan). For the analysis, lyophilized samples were fixed onto a sample holder and gold-coated for 90 s.

#### 3.3.2. Dynamic Light Scattering (DLS) and Zeta Potential

Dynamic Light Scattering (DLS) was used to determine the size of NPs as well as the polydispersity index (PDI) employing a DynaPro MS/X, (Wyatt Inc., Santa Barbara, CA, USA). Dry nanoparticles were re-suspended in deionized water and sonicated for 10 min to improve dispersion homogeneity. Every sample was housed in quartz cuvettes and measured 40 times in one run. The zeta potential values were determined by laser Doppler anemometry using a Zetasizer Nano ZS instrument (Malvern, UK) at 25 °C. Samples were measured in triplicate.

### 3.4. Encapsulation Efficiency (EE) and Drug-Loading Capacity (LC) Estimations

The encapsulation efficiency and loading capacity of S14-loaded NPs were determined by a direct method with HPLC analysis [28]. The samples were analyzed in a SunFire^®^ C18, 3.5 μm, 4.6 × 50 mm^2^ column (Waters, Cerdanyola del Vallés, Spain) and UV-Vis spectra were acquired using a Thermo Finnigan Surveyor UV-Vis Plus Detector (ThermoFisher, Madrid, Spain). The entrapped drug (S14*_entrapped_*) was detected by direct injection of the samples after breaking the nanoparticles as follows: 2.5 mg of NPs were dissolved in 5 mL of acetonitrile and the mixture was sonicated for 25 min. Then, 1 mL of solution was filtered over a 0.22 μm filter (Minisart^®^ Syringe Filter, Polietersulfona (PES), ethylene oxide, Cat.#16532-K) and analyzed in the HPLC system. The encapsulation efficiency and drug-loading capacity values were expressed according to the following Equations:

EE% = (S14*_entrapped_*/S14*_total_*) × 100 (1)

LC% = (S14*_entrapped_*/NP*s_total_*) × 100 (2)

For the calibration curve, six standard solutions at concentrations ranging from 0.2 mM to 0.7 mM of free S14 were prepared and analyzed under the same conditions.

### 3.5. Cell Culture and Cell Viability

Human neuroblastoma SH-SY5Y cell line was cultured in Dulbecco’s Modified Eagle Medium (DMEM, Gibco) supplemented with 10% fetal bovine serum (FBS, Gibco) and 1% penicillin-streptomycin (Gibco) at 37 °C and 5% CO_2_. Cell viability of SH-SY5Y cells exposed to different concentrations of free S14 or S14-loaded nanoparticles NP-2.6 and NP-2.8 for 24 h was determined by the 3-(4,5-dimethylthiazol-2-yl)-2,5-diphenyl tetrazolium bromide (MTT) assay. Sixty thousand cells were seeded onto 96-well plates and treated with different concentrations of free S14 and S14-loaded nanoparticles (5 µM, 1 µM, 0.5 µM and 0.1 µM). Twenty-four h after the treatment, thiazolyl blue was added to the culture media at a final concentration of 0.25 mg/mL for at least 2 h at 37 °C. After the incubation, culture media was removed and formazan crystals were dissolved with 200 µL of DMSO. Absorbance at 595 nm was measured with a microplate reader (Varioskan Flash Microplate reader, Thermo Scientific, Waltham, MA, USA).

### 3.6. Pharmacokinetic Studies

Healthy male Swiss Albino mice (8–12 weeks old) weighing between 25 and 35 g were used in the study. A total of 54 mice were used: 27 per oral administration of free S14 and 27 per S14-loaded nanoparticles (NP-2.5). In both cases, the formulation was based in phosphate buffer saline (PBS, pH 7.4) at a dose of 10 mg/kg. Blood samples (≈60 μL) were collected from a set of three mice at each time point (pre-dose, 0.25, 0.5 1, 2, 4, 6, 8 and 24 h). Immediately after collection of blood, brain samples were collected from set bioanalysis. Concentrations of compound S14 in mouse plasma and brain samples were determined by fit-for-purpose LC–MS/MS method.

## 4. Conclusions

In this work, PLGA-based biodegradable polymeric nanoparticles were synthetized, optimized and characterized for the encapsulation of S14, a potent PDE7 inhibitor, as a novel strategy for the treatment of Parkinson’s disease. The evaluation of the S14-loaded PLGA-NPs both in vitro and in vivo has demonstrated not only the safety but also the efficacy of these nanocarriers to improve the pharmacokinetic properties of S14, showing its great potential for the clinical translation of this interesting drug. Preliminary laboratory scale-up studies demonstrate that S14-loaded nanoparticles may be a good new strategy to improve the pharmacokinetic profile of this PDE7 inhibitor, boosting its advance for clinical applications.

## Figures and Tables

**Table 1:** Composition of the different PLGA nanoparticles prepared using the single-emulsion method.

Formulations	Raw Materials					Final NPs (mg)
	S14 (mg)	Polymer Type	Polymer Concentration (% *w*/*v*) ^a^	Surfactant Type ^b^	Surfactant Concentration (% *w*/*v*)	
NP-1.1	10.3	PLGA 50:50	1.25	CNC	0.5	10.0
NP-1.2	10.0	PLGA 50:50	1.25	P188	1	n.o. *
NP-1.3	10.2	PLGA 50:50	1.25	P188	2	n.o. *
NP-1.4	10.0	PLGA 50:50	1.25	P188	5	n.o. *
NP-1.5	10.4	PLGA 50:50	1.25	PVA	2	11.0
NP-1.6	20.5	PLGA 50:50	1.25	PVA	2	27.4

^a^ Ethyl acetate (EtOAc) was used as organic solvent; ^b^ CNC: cellulose nanocrystals, P188: poloxamer 188, PVA: polyvinyl alcohol.; * n.o.: not obtained.

**Table 2:** Composition of PLGA nanoparticles prepared by nanoprecipitation methodology.

Formulations	Raw Materials					Final NPs (mg)
	S14 (mg)	Polymer Type	Polymer Concentration (% *w*/*v*) ^a^	Surfactant Type ^b^	Surfactant Concentration (% *w*/*v*)	
NP-2.1	10.3	PLGA 50:50	0.5	PVA	2	36.3
NP-2.2	10.1	PLGA 50:50	0.5	PVA	2	32.7
NP-2.3	20.0	PLGA 50:50	0.5	PVA	2	55.0
NP-2.4	20.2	PLGA 50:50	0.5	PVA	2	55.5
NP-2.5	30.0	PLGA 50:50	0.5	PVA	2	53.6
NP-2.6	30.1	PLGA 50:50	0.5	PVA	2	59.4
NP-2.7	40.0	PLGA 50:50	0.5	PVA	2	74.3
NP-2.8	40.5	PLGA 50:50	0.5	PVA	2	78.7

^a^ Tetrahydrofuran (THF) was used as organic solvent;^. b^ PVA: polyvinyl alcohol.

**Table 3:** Encapsulation efficiency and drug-loading capacity of S14-loaded PLGA nanoparticles prepared by duplicate using nanoprecipitation methodology.

Formulations	Initial S14 (mg)	Final NPs (mg)	S14 Encapsulated (mg)	EE%	LC%
NP-2.1	10.3	36.3	7.3	71	20
NP-2.2	10.1	32.7	6.6	65	20
NP-2.3	20.0	55.0	15.7	78	28
NP-2.4	20.2	55.5	14.5	72	26
NP-2.5	30.0	53.6	21.6	72	40
NP-2.6	30.1	59.4	21.3	71	36
NP-2.7	40.0	74.3	35.2	88	47
NP-2.8	40.5	78.7	35.2	87	45

**Table 4:** Escalation process of S14-loaded PLGA nanoparticles by nanoprecipitation method.

Formulations	Raw Materials					Final NPs (mg)
	S14 (mg)	Polymer Type	Polymer Concentration (% *w*/*v*) ^a^	Surfactant Type ^b^	Surfactant Concentration (% *w*/*v*)	
NP-3.1	200.3	PLGA 50:50	0.5	PVA	2	239.0
NP-3.2	202.4	PLGA 50:50	0.5	PVA	2	241.8
NP-3.3	200.3	PLGA 50:50	0.5	PVA	2	224.0
NP-3.4	202.0	PLGA 50:50	0.5	PVA	2	249.2
NP-3.5	203.5	PLGA 50:50	0.5	PVA	2	231.4

^a^ Tetrahydrofuran (THF) was used as organic solvent; ^b^ PVA: polyvinyl alcohol.

## Data Availability

The data presented in this study are available in the article and supplementary material and on request from the corresponding author.

## Supplementary Materials

The following are available online at https://www.mdpi.com/1422-0067/22/6/3206/s1, Figure S1: HPLC analysis. Linear correlation between the area under the curve and the concentration of free S14, Figure S2: Representative SEM image (a) and size distribution (b) of S14-loaded PLGA nanoparticles after the escalation process (NP-3.1). DLS results are shown as the mean of 40 measures ± standard deviation (SD). PDI refers to polydispersity of NPs. Table S1: Pharmacokinetic studies in mice. S14-loaded PLGA-NPs (NP-2.5) and Free S14 concentrations after oral administration. Table S2: Encapsulation efficiency and drug-loading capacity of S14-loaded PLGA nanoparticles after the escalation process.

## References

[1] Francis S.H., Blount M.A., Corbin J.D. (2011). Mammalian cyclic nucleotide phosphodiesterases: Molecular mechanisms and physiological functions. Physiol. Rev..

[2] Martinez A., Gil C. (2014). cAMP-specific phosphodiesterase inhibitors: Promising drugs for inflammatory and neurological diseases. Expert Opin. Ther. Pat..

[3] García A.M., Brea J., Morales-García J.A., Perez D.I., González A., Alonso-Gil S., Gracia-Rubio I., Ros-Simó C., Conde S., Cadavid M.I. (2014). Modulation of cAMP-specific PDE without emetogenic activity: New sulfide-like PDE7 inhibitors. J. Med. Chem..

[4] Bales K.R., Menniti F.S., Martinez A. (2010). Promoting synaptic resilience in Alzheimer’s disease patients through phosphodiesterases inhibition. Emerging Drugs and Targets for Alzheimer’s Disease: Volume 2: Neuronal Plasticity.

[5] Martinez A., Gil C., Martinez A., Gil C. (2013). Phosphodiesterase inhibitors as new therapeutic approach for the treatment of Parkinson’s disease. Emerging Drugs and Targets for Parkinson’s Disease.

[6] Morales-Garcia J.A., Alonso-Gil S., Santos A., Perez-Castillo A. (2020). Phosphodiesterase 7 regulation in cellular and rodent models of Parkinson’s disease. Mol. Neurobiol..

[7] Morales-Garcia J.A., Aguilar-Morante D., Hernandez-Encinas E., Alonso-Gil S., Gil C., Martinez A., Santos A., Perez-Castillo A. (2015). Silencing phosphodiesterase 7B gene by lentiviral-shRNA interference attenuates neurodegeneration and motor deficits in hemiparkinsonian mice. Neurobiol. Aging.

[8] Jankowska A., Swierczek A., Chlon-Rzepa G., Pawlowski M., Wyska E. (2017). PDE7-Selective and dual inhibitors: Advances in chemical and biological research. Curr. Med. Chem..

[9] Castano T., Wang H., Campillo N.E., Ballester S., Gonzalez-Garcia C., Hernandez J., Perez C., Cuenca J., Perez-Castillo A., Martinez A. (2009). Synthesis, structural analysis, and biological evaluation of thioxoquinazoline derivatives as phosphodiesterase 7 inhibitors. ChemMedChem.

[10] Morales-Garcia J.A., Redondo M., Alonso-Gil S., Gil C., Perez C., Martinez A., Santos A., Perez-Castillo A. (2011). Phosphodiesterase 7 inhibition preserves dopaminergic neurons in cellular and rodent models of Parkinson disease. PLoS ONE.

[11] Morales-Garcia J.A., Echeverry-Alzate V., Alonso-Gil S., Sanz-SanCristobal M., Lopez-Moreno J.A., Gil C., Martinez A., Santos A., Perez-Castillo A. (2017). Phosphodiesterase7 inhibition activates adult neurogenesis in hippocampus and subventricular zone in vitro and in vivo. Stem Cells.

[12] Morales-Garcia J.A., Alonso-Gil S., Gil C., Martinez A., Santos A., Perez-Castillo A. (2015). Phosphodiesterase 7 inhibition induces dopaminergic neurogenesis in hemiparkinsonian rats. Stem Cells Transl. Med..

[13] Patra J.K., Das G., Fraceto L.F., Campos E.V.R., Rodriguez-Torres M.D.P., Acosta-Torres L.S., Diaz-Torres L.A., Grillo R., Swamy M.K., Sharma S. (2018). Nano based drug delivery systems: Recent developments and future prospects. J. Nanobiotechnol..

[14] Anselmo A.C., Mitragotri S. (2019). Nanoparticles in the clinic: An update. Bioeng. Transl. Med..

[15] Joshi S.A., Chavhan S.S., Sawant K.K. (2010). Rivastigmine-loaded PLGA and PBCA nanoparticles: Preparation, optimization, characterization, in vitro and pharmacodynamic studies. Eur. J. Pharm. Biopharm..

[16] Mansour H.M., Sohn M., Al-Ghananeem A., Deluca P.P. (2010). Materials for pharmaceutical dosage forms: Molecular pharmaceutics and controlled release drug delivery aspects. Int. J. Mol. Sci..

[17] Makadia H.K., Siegel S.J. (2011). Poly Lactic-co-Glycolic Acid (PLGA) as biodegradable controlled drug delivery carrier. Polymers.

[18] Crucho C.I.C., Barros M.T. (2017). Polymeric nanoparticles: A study on the preparation variables and characterization methods. Mater. Sci. Eng. C.

[19] Wischke C., Schwendeman S.P. (2008). Principles of encapsulating hydrophobic drugs in PLA/PLGA microparticles. Int. J. Pharm..

[20] Caldas Dos Santos T., Rescignano N., Boff L., Reginatto F.H., Simões C.M.O., de Campos A.M., Mijangos C. (2017). In vitro antiherpes effect of C-glycosyl flavonoid enriched fraction of Cecropia glaziovii encapsulated in PLGA nanoparticles. Mater. Sci. Eng. C.

[21] Yan F., Zhang C., Zheng Y., Mei L., Tang L., Song C., Sun H., Huang L. (2010). The effect of poloxamer 188 on nanoparticle morphology, size, cancer cell uptake, and cytotoxicity. Nanomedicine.

[22] Halib N., Perrone F., Cemazar M., Dapas B., Farra R., Abrami M., Chiarappa G., Forte G., Zanconati F., Pozzato G. (2017). Potential applications of nanocellulose-containing materials in the biomedical field. Materials.

[23] Sahoo S.K., Panyam J., Prabha S., Labhasetwar V. (2002). Residual polyvinyl alcohol associated with poly (D,L-lactide-co-glycolide) nanoparticles affects their physical properties and cellular uptake. J. Control. Release.

[24] Zhou W., Wang Z.L. (2007). Scanning Microscopy for Nanotechnology.

[25] Fessi H., Puisieux F., Devissaguet J.P., Ammoury N., Benita S. (1989). Nanocapsule formation by interfacial polymer deposition following solvent displacement. Int. J. Pharm..

[26] Rojas-Prats E., Tosat-Bitrian C., Martinez-Gonzalez L., Nozal V., Perez D.I., Martinez A. (2021). Increasing brain permeability of PHA-767491, a cell division cycle 7 kinase inhibitor, with biodegradable polymeric nanoparticles. Pharmaceutics.

[27] Shen S., Wu Y., Liu Y., Wu D. (2017). High drug-loading nanomedicines: Progress, current status, and prospects. Int. J. Nanomed..

[28] Amini Y., Amel Jamehdar S., Sadri K., Zare S., Musavi D., Tafaghodi M. (2017). Different methods to determine the encapsulation efficiency of protein in PLGA nanoparticles. Biomed. Mater. Eng..

[29] Danhier F., Ansorena E., Silva J.M., Coco R., Le Breton A., Préat V. (2012). PLGA-based nanoparticles: An overview of biomedical applications. J. Control. Release.

[30] Carvalho P.M., Felício M.R., Santos N.C., Gonçalves S., Domingues M.M. (2018). Application of light scattering techniques to nanoparticle characterization and development. Front. Chem..

[31] Kreuter J. (2014). Drug delivery to the central nervous system by polymeric nanoparticles: What do we know?. Adv. Drug Deliv. Rev..

[32] Rescignano N., Fortunati E., Armentano I., Hernandez R., Mijangos C., Pasquino R., Kenny J.M. (2015). Use of alginate, chitosan and cellulose nanocrystals as emulsion stabilizers in the synthesis of biodegradable polymeric nanoparticles. J. Colloid Interface Sci..

[33] Pahuja R., Seth K., Shukla A., Shukla R.K., Bhatnagar P., Chauhan L.K., Saxena P.N., Arun J., Chaudhari B.P., Patel D.K. (2015). Trans-blood brain barrier delivery of dopamine-loaded nanoparticles reverses functional deficits in parkinsonian rats. ACS Nano.

[34] Martinez A., Gil C., Lopez-Ribas D., Cunchillos E., Domenech J., Sarasa M. (2016). Targeting PDE7 by the small molecule S14: A potential disease-modifying Parkinson’s disease therapy ready to start clinical trials. Movement Disorders.

