# Supplementary material for: Improved Controlled Release and Brain Penetration of the Small Molecule S14 Using PLGA Nanoparticles
Source: Int J Mol Sci. 2021 Mar 22;22(6):3206. doi: 10.3390/ijms22063206 (PMC8004175; doi:10.3390/ijms22063206)
Supplement: Supplementary file 1 [file ijms-22-03206-s001.pdf]

International Journal of
Molecular Sciences

MDPI

Article

Improved Controlled Release and Brain Penetration of the Small Molecule S14 Using PLGA Nanoparticles †

Vanesa Nozal [1,‡], Elisa Rojas-Prats [1,‡], Inés Maestro [1], Carmen Gil [1,2], Daniel I. Perez [1,*] and Ana Martinez [1,2,*]

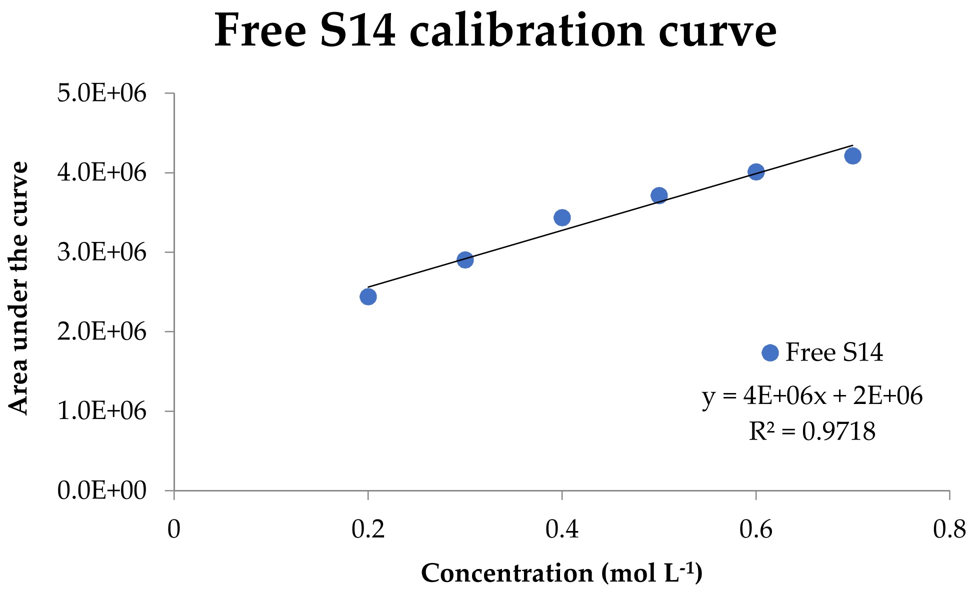

Figure S1. HPLC analysis. Linear correlation between the area under the curve and the concentration of free S14.

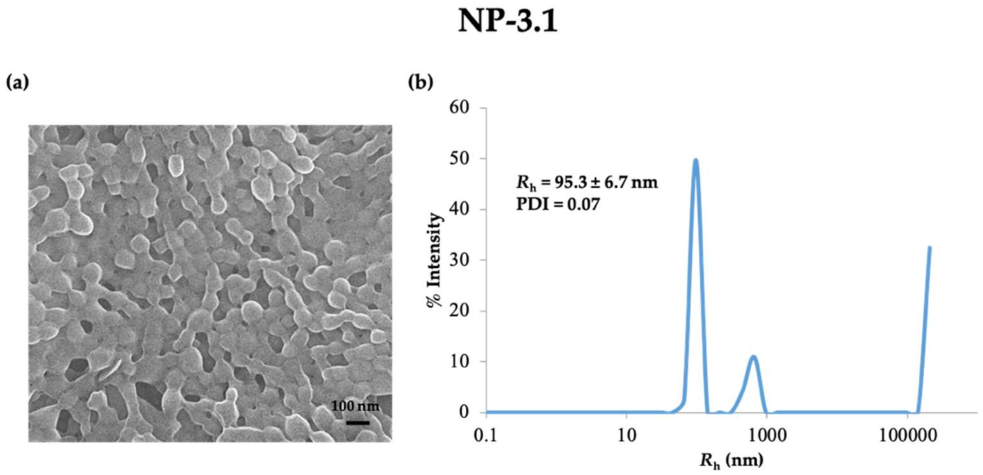

Figure S2. Representative SEM image (a) and size distribution (b) of S14-loaded PLGA nanoparticles after the escalation process (NP-3.1). DLS results are shown as the mean of 40 measures ± standard deviation (SD). PDI refers to polydispersity of NPs.

Table S1. Pharmacokinetic studies in mice. S14 loaded PLGA-NPs (NP-2.5) and Free S14 concentrations after oral administration.

Formulation	Dose (mg/kg)	Time (hours)	Plasma Conc. (ng/mL)	Brain Conc. (ng/g)	Plasma-to-brain ratio
NP-2.5	10	Predose	0.00	0.00	-
		0.25	109.44 ± 45.42	115.65 ± 86.93	0.95
		0.5	213.06 ± 30.82*	129.66 ± 7.09*	1.64
		1	204.82 ± 55.23	105.19 ± 40.20	1.95
		2	462.18 ± 54.03	226.93 ± 20.37	2.04
		4	248.43 ± 75.95	157.36 ± 42.13	1.58
		6	132.86 ± 93.29	74.77 ± 47.05	1.78
		8	32.31 ± 5.74	16.06 ± 3.71	2.01
		24	0.00	0.00	-
Free S14	10	Predose	0.00	0.00	-
		0.25	1173.70 ± 113.76	300.66 ± 32.71	3.90
		0.5	682.91 ± 108.24	179.36 ± 40.07	3.81
		1	405.29± 107.10	95.41 ± 26.25	4.25
		2	119.30 ± 37.66	27.75 ± 5.71	4.30
		4	40.37 ± 24.70	11.05 ± 5.33	3.65
		6	16.61 ± 3.73	5.45 ± 1.12	3.05
		8	14.69 ± 6.27	1.85 ± 3.20	7.95
		24	5.70 ± 6.08	1.30 ± 2.25	4.39

*Mean of two values considered for data analysis

Table S2. Encapsulation efficiency and drug-loading capacity of S14-loaded PLGA nanoparticles after the escalation process.

Formulations	Initial S14 (mg)	Final NPs (mg)	S14 encapsulated (mg)	EE%	LC%
NP-3.1	200.3	239.0	140.2	70%	59%
NP-3.2	202.4	241.8	133.6	66%	55%
NP-3.3	200.3	224.0	132.2	66%	59%
NP-3.4	202.0	249.2	147.5	73%	59%
NP-3.5	203.5	231.4	120.1	59%	52%
